# Rapid synchronized fabrication of vascularized thermosets and composites

**DOI:** 10.1038/s41467-021-23054-7

**Published:** 2021-05-14

**Authors:** Mayank Garg, Jia En Aw, Xiang Zhang, Polette J. Centellas, Leon M. Dean, Evan M. Lloyd, Ian D. Robertson, Yiqiao Liu, Mostafa Yourdkhani, Jeffrey S. Moore, Philippe H. Geubelle, Nancy R. Sottos

**Affiliations:** 1 Beckman Institute for Advanced Science and Technology, University of Illinois at Urbana-Champaign, Urbana, IL USA; 2 Departments of Materials Science and Engineering, University of Illinois at Urbana-Champaign, Urbana, IL USA; 3 Department of Aerospace Engineering, University of Illinois at Urbana-Champaign, Urbana, IL USA; 4 Department of Chemical and Biomolecular Engineering, University of Illinois at Urbana-Champaign, Urbana, IL USA; 5 Department of Chemistry, University of Illinois at Urbana-Champaign, Urbana, IL USA; 6 Present Address: Department of Mechanical Engineering, University of Wyoming, Laramie, WY USA; 7 Present Address: Department of Mechanical Engineering, Colorado State University, Fort Collins, CO USA

**Keywords:** Gels and hydrogels, Gels and hydrogels, Mechanical properties

## Abstract

Bioinspired vascular networks transport heat and mass in hydrogels, microfluidic devices, self-healing and self-cooling structures, filters, and flow batteries. Lengthy, multistep fabrication processes involving solvents, external heat, and vacuum hinder large-scale application of vascular networks in structural materials. Here, we report the rapid (seconds to minutes), scalable, and synchronized fabrication of vascular thermosets and fiber-reinforced composites under ambient conditions. The exothermic frontal polymerization (FP) of a liquid or gelled resin facilitates coordinated depolymerization of an embedded sacrificial template to create host structures with high-fidelity interconnected microchannels. The chemical energy released during matrix polymerization eliminates the need for a sustained external heat source and greatly reduces external energy consumption for processing. Programming the rate of depolymerization of the sacrificial thermoplastic to match the kinetics of FP has the potential to significantly expedite the fabrication of vascular structures with extended lifetimes, microreactors, and imaging phantoms for understanding capillary flow in biological systems.

## Abstract (web summary)

Bioinspired vascular networks transport heat and mass in multifunctional materials but lengthy multistep fabrication processes hinder large-scale application of structural vascular materials. Here, the authors report rapid, scalable, and synchronized fabrication of vascular thermosets and fiberreinforced composites under ambient conditions.

## Introduction

Biological materials possess hierarchical vascular networks that mediate heat and mass transport in response to external and internal stimuli, enabling complex living systems to thrive in extreme environments^1,2^. The replication of such pervasive vascular networks in engineered tissues and organoids for improving cellular proliferation^3–5^, studying fluid flow^6^, and pneumatic actuation^7^ has garnered significant interest in therapeutics, prosthetics, and soft robotics^3–6^. Vascular and porous synthetic materials are also used for electrical insulation in microelectronics^8^, gas exchange in synthetic devices^9,10^, thermal management in heat exchangers and fiber-reinforced composites^11,12^, chemical reactions in flow batteries and microreactors^13–15^, and material regeneration in self-healing structures^16,17^.

The new vasculature in biological load-bearing materials such as bone is created through coordinated deposition of new tissue and removal of old tissue, triggered by coupled cellular processes^18–20^. In contrast, the formation of vasculature inside synthetic materials requires an additional fabrication step to remove embedded sacrificial templates through pullout^17,21^, dissolution^3,6,22–25^, melting^26–29^, or thermal vaporization^16,30–35^ techniques. Vaporization of sacrificial components (VaSC) is the only method that enables the fabrication of complex vascular networks in load-bearing polymers and composites processed at high temperatures and pressures^16,30,31,33^. However, the VaSC process requires a sustained external heat source first to cure the host matrix and subsequently to depolymerize the sacrificial template (ca. 200 °C for 12–24 h under vacuum)^31^, consuming megajoules to gigajoules of external energy^36^. New strategies for faster matrix curing and template depolymerization at lower temperatures are thus desirable to reduce the time, energy, and complexity of fabricating vascular structures for aerospace, automotive, marine, biomedical, and renewable energy industries.

## Results and discussion

### Material system for tandem polymerization and vascularization

**Fig. 1:**
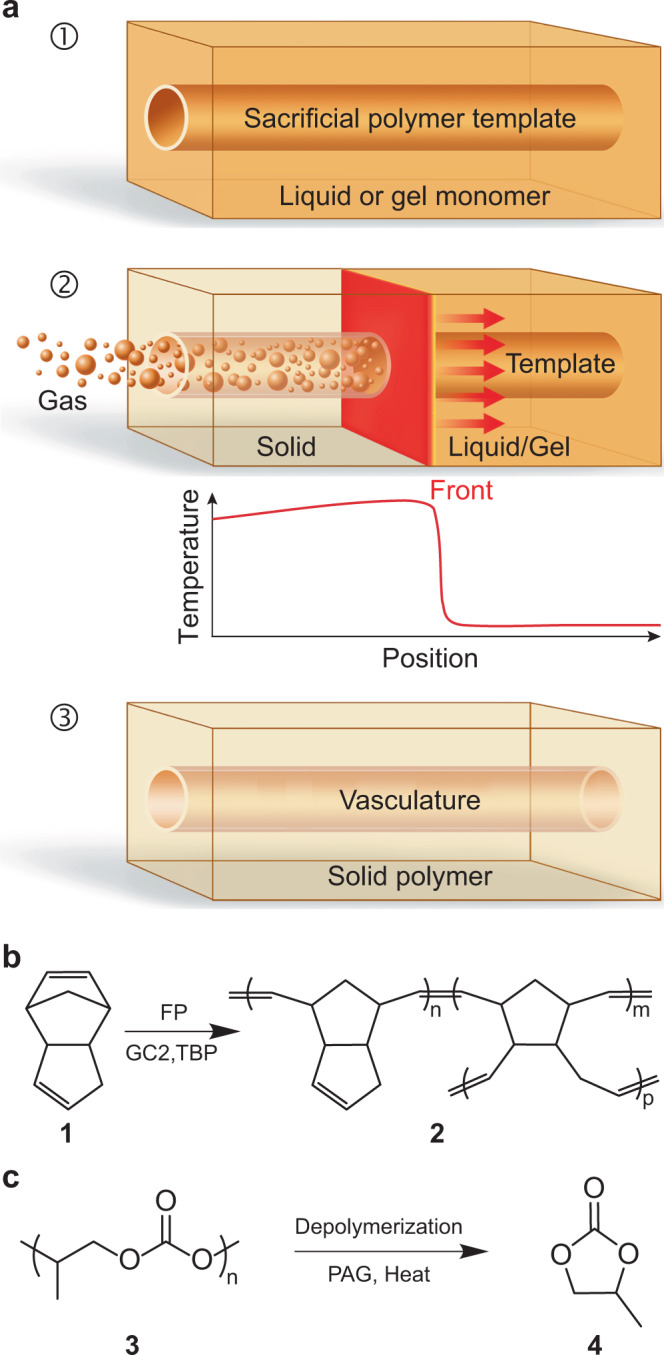
Overview of synchronized polymerization and vascularization concept. **a** Schematic representation of the coordinated fabrication of a microvascular structure. ➀A sacrificial polymer template is embedded inside a liquid or gelled host matrix. ➁A self-propagating exothermic polymerization reaction transforms the host into a solid matrix and concurrently depolymerizes the sacrificial template into small molecules through a localized increase in temperature. ➂A durable thermoset with vasculature mirroring the starting template is manufactured under standard ambient conditions within minutes. **b** Scheme for FP of DCPD monomer (**1**) into crosslinked pDCPD (**2**) using a second-generation Grubbs’ catalyst (GC2) and tributyl phosphite inhibitor (TBP). **c** Scheme for photoacid generator (PAG) catalyzed thermal depolymerization of PPC (**3**) into PC monomer (**4**).

Inspired by the coordinated vascularization process in biological structures, we have developed a coupled polymerization and depolymerization strategy for rapid manufacturing of vascular thermosets and composites under ambient conditions (Fig. 1a). This synchronized process relies on the exothermic frontal polymerization (FP)^37,38^ of the dicyclopentadiene (DCPD) monomer. Here, a sacrificial template is first embedded in liquid or gelled DCPD resin. Brief (seconds), localized heating activates a latent second-generation Grubbs’ catalyst (GC2) to initiate the self-propagating reaction wave that converts a liquid or gelled resin into rigid poly(dicyclopentadiene) (pDCPD) thermoset (Fig. 1b). The surplus of energy released during the curing reaction is harnessed for concurrent depolymerization of an embedded sacrificial template. This tandem approach eliminates the need for a sustained external heat source to manufacture vascular structures, enabling efficient fabrication at room temperature (RT).

Successful coordination between polymerization and vascularization requires a sacrificial polymer that remains stable during melt processing and integration into the host matrix, yet depolymerizes efficiently and rapidly under the thermal conditions generated during FP. In situ temperature measurements during FP of DCPD resin incubated to different degrees of precure (*α*_0_) show maximum temperatures exceeding 200 °C for *α*_0_ ≤ 0.08, followed by cooling to below 100 °C within 2 min (Supplementary Fig. 1c). The chemical enthalpy released during FP decreases with increasing *α*_0_ (Eq. 1 in the “Methods” section), thus reducing the maximum temperature. Thermal vaporization of poly(lactic acid) (PLA) templates used in prior vascularization methods requires several hours at such temperatures^30,31^, making PLA an unsuitable sacrificial polymer for our strategy. We found that these seemingly incompatible requirements for high-temperature processing and low-temperature depolymerization are satisfied by poly(propylene carbonate) (PPC)^39^. In its thermally stable state, PPC is templated into fibers, sheets, and printed architectures. An orthogonal stimulus such as UV light subsequently transforms the template into a thermally unstable state for facile depolymerization.

PPC is thermally stable up to 250 °C but undergoes acid-catalyzed depolymerization into propylene carbonate (PC) monomer (Fig. 1c) at much lower temperatures (100–200 °C)^39^. Melt extrusion of PPC blended with an acid catalyst is not feasible due to depolymerization at typical processing temperatures (150–200 °C). Instead, the high thermal stability of a diaryliodonium-based photoacid generator (PAG) (up to ca. 180 °C^39^) enables the melt-spinning of PPC fibers (*M*_w_ ~ 196 kDa) blended with PAG at 155 °C without significant depolymerization (Supplementary Fig. 2a). The depolymerization onset temperature (*T*_d_, defined by 5% mass loss) of as-spun PPC (1% PAG) fibers occurs at 200 °C with complete mass loss at 230 °C (Supplementary Fig. 2b). Activation of the PAG via UV irradiation reduces *T*_d_ to ca. 93 °C with complete mass loss at 130 °C, which is ca. 100 °C lower than as-spun fibers. These mass loss results suggest that UV-irradiated PPC (1% PAG) templates are suitable for depolymerization during FP of the host matrix.

### Successful vascularization window in neat thermosets

**Fig. 2:**
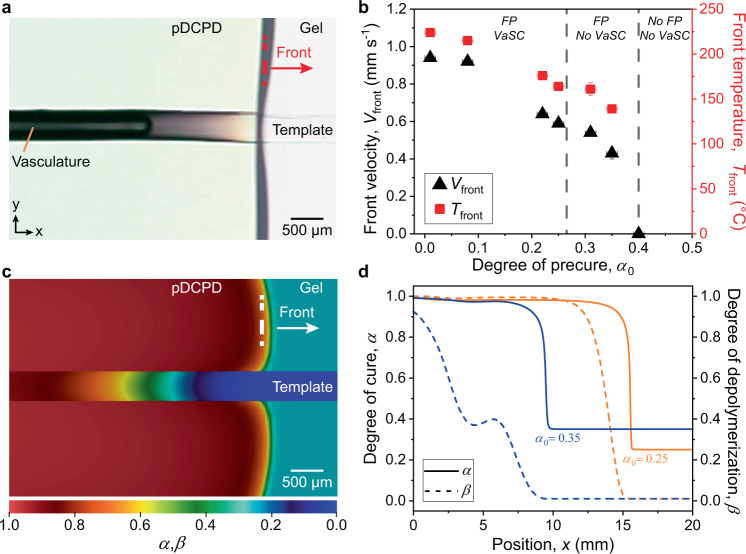
Experimental characterization and thermochemical modeling of the coordinated vascularization process. **a** Optical image of propagating FP reaction in a DCPD gel (*α*_0_ = 0.25) in tandem with depolymerization of an embedded PPC (1% PAG) template to create vascular pDCPD (*t* = 31 s after initiation). **b** Front velocity (black triangles) and maximum front temperature (red squares) during FP as a function of *α*_0_ of the DCPD gel. Successful VaSC through complete depolymerization of PPC depends on the amount of heat released during FP, which decreases with increasing *α*_0_. Sacrificial templates undergo complete depolymerization for *α*_0_ ≤ 0.25. Partial depolymerization results in clogged microchannels for 0.30 ≤ *α*_0_ ≤ 0.35 and FP is no longer possible for *α*_0_ ≥ 0.40. Error bars represent one standard deviation from the mean (*n* = 3). **c** Simulation of a DCPD gel (*α*_0_ = 0.25) showing the spatial distribution of the degree of cure (*α*) of DCPD and the degree of depolymerization (*β*) of the sacrificial template during FP (*t* = 31 s after initiation). **d** Predicted spatial variation of *α* and *β* in the direction of the propagating front (*t* = 31 s after initiation). Successful polymerization and vascularization are defined by *α* and *β* reaching 0.90 or higher, as shown by the *α*_0_ = 0.25 case (orange lines). The *α*_0_ = 0.35 case (blue lines) shows successful polymerization but unsuccessful vascularization, corroborating experimental observations.

Initially, we investigated the depolymerization of a single UV-irradiated PPC (1% PAG) fiber in tandem with FP of a DCPD gel (*α*_0_ = 0.25) (Fig. 2a). The sacrificial fiber is embedded in liquid resin which cures slowly at RT into a gel with the desired *α*_0_ (Supplementary Table 1). FP is triggered by briefly powering a resistive wire oriented perpendicular to the fiber. The polymerization front propagates through the specimen at ca. 0.6 mm s^−1^ and vascularization trails by ca. 1.5 mm (Fig. 2a, Supplementary Video 1). A slight distortion in the shape of the polymerization front also occurs near the sacrificial fiber. Temporal delays associated with heat conduction from the matrix to the template and its subsequent endothermic depolymerization into liquid and gaseous molecules are responsible for these observations. Optical microscopy of the microchannel cross section and X-ray computed microtomography (μCT) reconstruction of the microchannel volume reveal that the dimensions and circularity of the channel replicate the sacrificial fiber (Supplementary Fig. 4 and Supplementary Table 5). As expected, as-spun fibers without UV irradiation do not depolymerize in similar experiments (Supplementary Fig. 5). Vascularization of pDCPD specimens with UV-irradiated sacrificial fibers occurs in less than 2 min under ambient conditions after triggering FP. Activation of the PAG and FP initiation require minimal energy input, ca. 108 J, reducing the thermal energy consumption by six orders of magnitude compared to ca. 120 MJ estimated for conventional curing and VaSC steps for samples of similar size (Supplementary Table 2).

The depolymerization kinetics of PPC is highly temperature-sensitive (Supplementary Fig. 6a, b) and successful vascularization depends on the surplus heat released by the host matrix during FP. Using the single sacrificial fiber geometry inside a glass mold, we explore the potential range of *α*_0_ for vascularization of the host matrix. As *α*_0_ increases, both the front velocity (*V*_front_) and maximum front temperature (*T*_front_) decrease (Fig. 2b) due to a reduction in the fraction of chemical bonds capable of ring-opening during FP^38^. Concurrent FP and vascularization occur for *α*_0_ ≤ 0.25, yielding fully evacuated channels. While the polymerization front still propagates in gels with 0.30 ≤ *α*_0_ ≤ 0.35, the released heat is insufficient for complete depolymerization of the sacrificial fiber, leaving partially to fully obstructed microchannels (Supplementary Fig. 8). The polymerization front does not propagate in gels with *α*_0_ ≥ 0.40 due to the reduced heat released during FP and heat loss to the glass mold.

### Numerical modeling of coupled polymerization and depolymerization

To gain insight into the tandem vascularization process, we developed a coupled thermochemical model for the thermal field (*T*), the degree of cure (*α*) of DCPD, and the degree of depolymerization (*β*) of PPC. The reaction-diffusion model^38^ was implemented in a transient, nonlinear finite-element solver (see “Methods” section). Successful curing and depolymerization are numerically defined by *α* ≥ 0.90 and *β* ≥ 0.90, respectively. The simulation shown in Fig. 2c predicts localized deceleration of the polymerization front near the sacrificial template and a spatial delay in vascularization with respect to the location of the front, which are observed experimentally in Fig. 2a. The computational results reveal that these observations are the consequence of the time required for heat to transfer from the cured pDCPD into the PPC template for endothermic depolymerization. The *V*_front_ and *T*_front_ predictions for different *α*_0_ also match closely with experiments in the presence of the glass molds (Supplementary Fig. 9). The model predicts successful curing and vascularization for *α*_0_ = 0.25, while also predicting unsuccessful vascularization due to incomplete depolymerization of PPC for *α*_0_ = 0.35 (Fig. 2d), as observed experimentally.

We further exploited this coupled thermochemical model to investigate the effect of a more insulating boundary condition (i.e., air) on the window for successful vascularization. Slightly higher *V*_front_ and *T*_front_ are predicted for an air boundary compared to a glass mold boundary (Supplementary Fig. 10). The lower convective heat loss to air compared to the conduction-driven heat loss to the glass mold allows longer heat retention in the matrix after FP, thereby extending the window for vascularization to *α*_0_ ≤ 0.35 and the window for front propagation to *α*_0_ ≤ 0.50. To compare these predictions with experiments, elastomeric DCPD gels with *α*_0_ > 0.20 containing sacrificial fibers are removed from the molds and subjected to FP. The *V*_front_ and *T*_front_ measurements along with the successful vascularization regime agree with the calculations from the model and the cooling profile of the matrix after FP is substantially slower (Supplementary Fig. 1c). The model successfully captures the thermochemical competition between the heat generated by the host matrix, the heat consumed by the depolymerizing fiber, and the heat lost to the surroundings, which determine the successful fabrication of vascular structures.

### Advanced manufacturing of microvascular structures

**Fig. 3:**
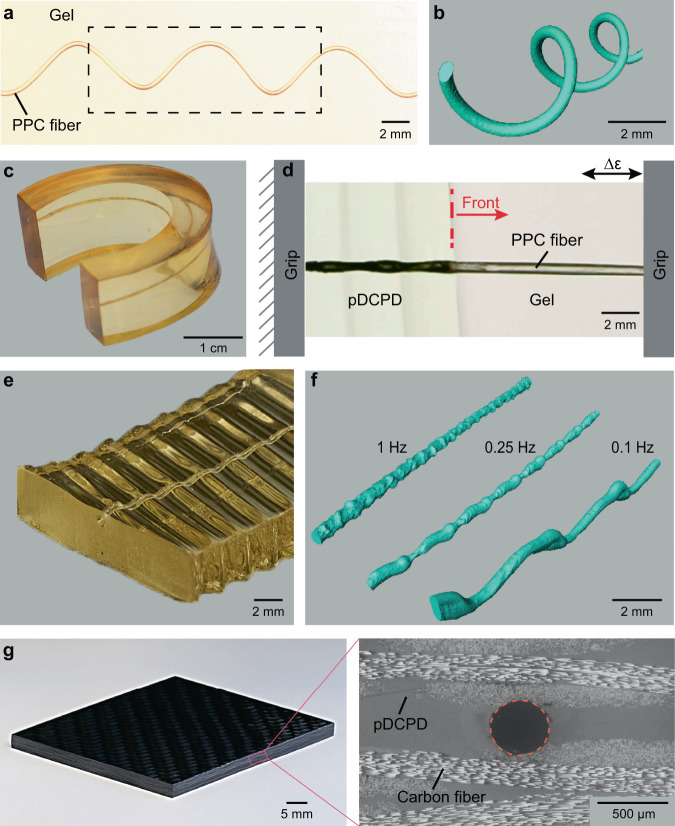
Advanced manufacturing of microvascular structures. **a**–**f** On-demand modulation of the vascular architecture in free-standing elastomeric DCPD gels (*α*_0_ = 0.25). **a** Optical image of a helical sacrificial template (diameter = 384 ± 10 μm) embedded in a DCPD gel prior to FP. **b** μCT reconstruction of the resulting microchannel (diameter = 404 ± 12 μm) filled with a radiocontrast fluid for the sample region encompassed by the dashed box in **a**. **c** Microchannel inside a horseshoe-shaped pDCPD structure traces the curvature of the matrix. **d**–**f** Concurrent polymerization and vascularization under oscillatory loading create sinusoidal microchannels. Representative optical images of FP under oscillatory strain (frequency = 0.25 Hz, amplitude = 4 mm) and resulting vascularized structure are shown in **d** and **e**, respectively. **f** µCT reconstructions of microchannels filled with a radiocontrast fluid show an increase in out-of-plane deformation for decreasing oscillation frequency. **g** Optical image and micrograph of the cross section of a void-free composite with a circular microchannel (dashed orange line) fabricated in 30 s under ambient conditions.

Our concurrent vascularization strategy offers excellent control over the matrix and microchannel architecture. Incubating the resin at RT to obtain viscoelastic gels proves advantageous for passive and dynamic modulation of the microchannel architecture. A range of complex vascular structures (Fig. 3a–f) is easily fabricated from elastomeric DCPD gels containing sacrificial PPC (1% PAG) templates. A 3D helical microchannel is created in pDCPD from a heat-set PPC template (Fig. 3a, b). μCT reconstruction of the microchannel reveals excellent dimensional and morphological fidelity. A flat gel with a straight fiber is bent into a horseshoe shape prior to FP without fracturing the template and this shape is preserved in the resulting microchannel (Fig. 3c). Further, this vascularization process also enables in situ modulation of the vascular geometry by applying oscillatory strain to a gel with a sacrificial template during FP (Fig. 3d and Supplementary Video 2). The polymerization front permanently sets the instantaneous out-of-plane deformation experienced by the gel and the fiber during each loading cycle (Fig. 3e). The pitch and amplitude of the channel are tuned by controlling the oscillation frequency (Fig. 3f). Such control over vascular features may find use in particle/cell separation^40^ or mixing of multiple fluids via non-laminar flow^26^.

Vascularized carbon-fiber-reinforced composites with circular microchannels are also rapidly manufactured with the FP-based method (Fig. 3g and Supplementary Table 5). The presence of the fiber reinforcement exacerbates the thermochemical competition between FP and VaSC processes due to the decreased resin volume, resulting in lower front temperatures^38^. Additional PAG is added to the PPC fibers to accelerate the depolymerization kinetics and achieve complete mass loss at lower temperatures (Supplementary Fig. 2c). PPC (3% PAG) fibers are sandwiched between layers of carbon-fiber fabric, then the fabric layup is infused with a liquid DCPD monomer. Briefly powering a resistive heating mat underneath the impregnated fabric stack triggers FP through the thickness of the laminate and facilitates longer heat retention in the cured composite, which ensures complete depolymerization of the sacrificial fiber. The fabrication time and energy are reduced by three and four orders of magnitude, respectively, compared to conventional curing and vascularization of similar size samples in an oven (Supplementary Table 2). We anticipate even more significant energy savings for larger components. For traditional autoclave cure and vaporization of templates at high temperature, energy consumption scales directly with part size^36,38^. In great contrast, the required energy input per unit volume for FP-mediated processing actually decreases with increasing component size^38^.

### Hierarchical interconnected vascular network

**Fig. 4:**
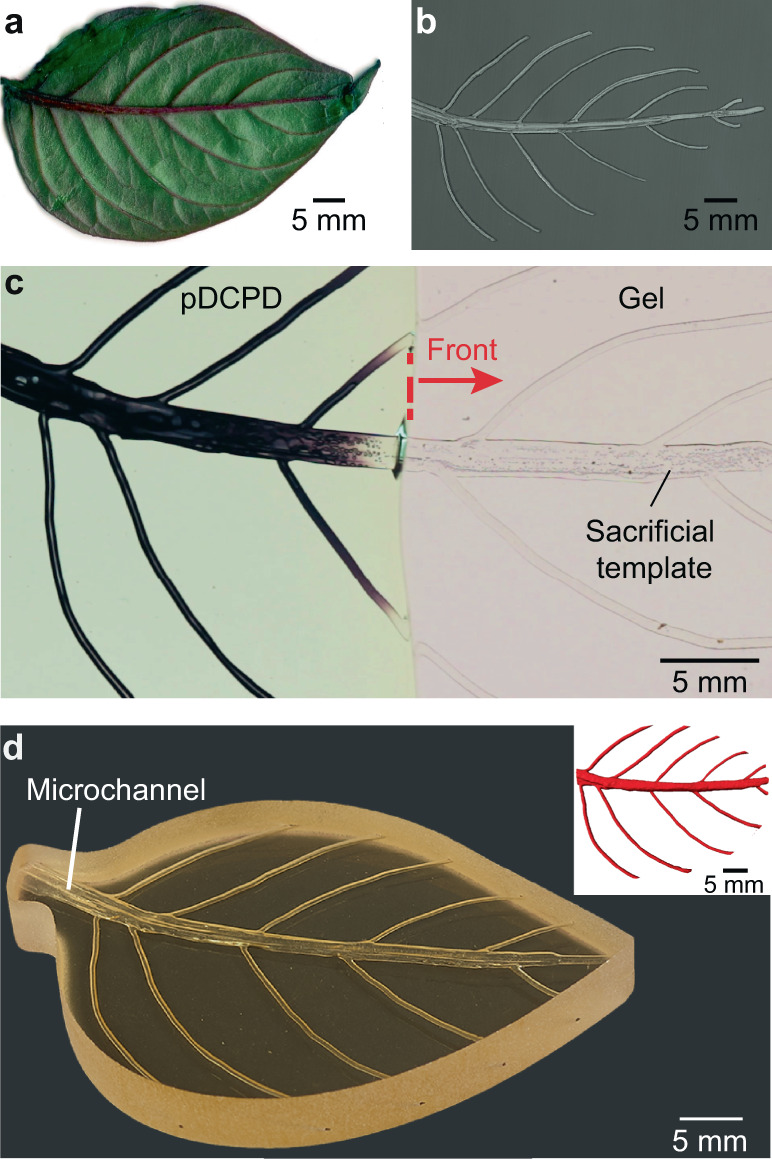
Synchronized manufacturing of a structure with hierarchical vascular network. **a** An *Impatiens* leaf. **b** 3D-printed PPC (1% PAG) template referencing veins of leaf in **a**. **c** Simultaneous polymerization and vascularization of DCPD gel (*α*_0_ = 0.25) with the embedded 3D-printed PPC (1% PAG) template. Depolymerization products escape through the central vein, completing the vascularization process in 80 s. **d** Optical image of the vascular structure and its µCT reconstruction (inset) show interconnectivity in the resulting microchannels.

The ability to 3D print sacrificial templates enables the fabrication of multiscale vascular structures with this tandem FP and vascularization strategy. A printed sacrificial PPC (1% PAG) template resembling the primary and secondary veins of an *Impatiens* leaf (Fig. 4a, b) is embedded in a DCPD gel (*α*_0_ = 0.25). During FP, the liquid and gaseous depolymerization products escape through the primary vein, forming interconnected microchannels behind the polymerization front (Fig. 4c). A vascular pDCPD structure with a 3× difference in width between the central and branched veins of the hollow network is formed in 80 s under ambient conditions (Supplementary Video 3). Optical micrograph and μCT reconstruction of the specimen in Fig. 4d reveal excellent replication of the hierarchical template.

Scaling of the vascularization process for larger structures and fabrication of more complex networks will require addressing several challenges. Currently, the architectural complexity is limited by the resolution and capability of the 3D printing process for the sacrificial templates. Our experiments and modeling reveal that the total heat available for depolymerization is dictated by the enthalpy of FP, the thermal conductance at the boundaries, the surrounding temperature, and the placement of the sacrificial templates. Templates that are placed near the surfaces of the host thermoset or composite will experience faster heat loss to the surroundings compared to the center, which could impede the formation of clear microchannels. Monomers with higher enthalpy of FP can increase the heat available for depolymerization. Alternatively, replacing metallic tooling surfaces with more thermally insulating materials such as rigid polyisocyanurate foams will prolong heat retention in large structures and aid complete depolymerization. The maximum size of a template and the irradiation time are dictated by the dimensions and the intensity of the UV lamp, respectively. We envision a narrowband LED source with a higher intensity for continuous irradiation of the fiber during spooling to circumvent these limitations. The maximum thickness of the templates that undergo complete depolymerization depends on sufficient activation of the PAG. Short-wave UV irradiation may not penetrate well through templates thicker than 600 μm, leading to an insufficient amount of activated PAG. Adding a photosensitizer to extend the absorption window to longer wavelengths will be beneficial in overcoming this challenge. The experimental methods and computational tools that we have developed can be used to investigate these different design parameters and help build a library of compatible host resins and sacrificial components for synchronized fabrication of large-scale vascular structures.

We have successfully exploited the frontal polymerization of a structural host matrix in tandem with the depolymerization of a sacrificial template to create a range of vascular structures in a rapid, energy-efficient single-step process. Excellent control over the microchannel shape, size, and complexity was achieved with orthogonally triggered sacrificial templates. Vascularization of thermosets with interconnected microchannels was achieved within a few minutes under ambient conditions with energy savings of six orders of magnitude over competing techniques. Our thermochemical model successfully captures the competition between the reaction enthalpy of the host matrix, the energy consumed during template depolymerization, and the energy dissipated into the surroundings. The model provides a promising tool for the design of host matrix and sacrificial components for FP-based synchronized manufacturing of vascular structures. Tandem polymerization and vascularization will propel the use of high-performance vascular structures by reducing the energetic and temporal footprint for fabrication by several orders of magnitude.

## Methods

### Materials

Dicyclopentadiene (DCPD), 5-ethylidene-2-norbornene (ENB), second-generation Grubbs’ catalyst (GC2), phenylcyclohexane (PCH), tributyl phosphite inhibitor (TBP), ethanol, and 5-(N-2,3-Dihydroxypropylacetamido)-2,4,6-triiodo-*N*,*N*′-bis(2,3-dihydroxypropyl)isophthalamide (Histodenz^TM^) are purchased from Sigma-Aldrich and used as received. Poly(propylene carbonate) (PPC) pellets are acquired from Novomer. 4-Methylphenyl[4-(1-methylethyl)phenyl]iodonium tetrakis(pentafluorophenyl)borate photoacid generator (PAG), commercially known as Rhodorsil-FABA, is gifted by Bluestar Silicones. The reinforcement for vascular composite specimens is Toray T300 carbon fiber 2 × 2 twill weave fabric. A 26-gauge Kanthal wire is used to trigger FP of neat resin specimens in glass molds. Post-FP, steel wires are used to verify channel clearance in neat and FRPC specimens.

### Resin formulation for frontal polymerization

Since DCPD is solid at 20 °C, we melt it in an oven at 40 °C and then add 5 wt.% ENB to depress the freezing point below room temperature (RT). For a typical experiment, the FP-capable resin is prepared by mixing 62.7 mg GC2 with 3.12 mL of PCH using a sonication bath. TBP (20 μL, i.e., one molar equivalent with respect to GC2) is added to the catalyst solution using a volumetric syringe. The GC2/PCH/TBP solution is then thoroughly mixed with 97.4 g DCPD/ENB solution (10,000 molar equivalents with respect to GC2). The resulting resin is used for experiments involving a neat matrix. For composite experiments, 0.3 molar equivalents of TBP are used. Unless otherwise specified, all FP experiments are performed under standard ambient conditions.

### Front temperature and velocity measurements

For both neat matrix and composite experiments, a T-type thermocouple (TMQSS, Omega) is inserted into the specimen midplane to measure the peak front temperature. The thermocouple is placed far away (ca. 15 mm) from embedded sacrificial fibers to avoid any influence on the polymerization front in the vicinity of the sacrificial fiber. The thermocouple is also far away from the resistive heating wire for temperature measurements during steady-state propagation of FP.

For the neat matrix experiments, a Canon EOS 7D digital camera with a 100 mm macro lens is focused on the plane of the sacrificial fiber embedded in the specimen. Tracker software (Open Source Physics) is used to determine the location of the polymerization front along the specimen length based on color and refractive index mismatch between the uncured and cured matrix in the optical images extracted from the Canon video. The front velocity is determined from the slope of the best-fit trendline for the position of the polymerization front as a function of time.

### Resin rheology

The storage and loss moduli of the DCPD resin as it incubates (slowly cures) under isothermal conditions (20 °C) are extracted from time sweep measurements at 0.1% strain and 1 Hz frequency on an AR-G2 rheometer (TA Instruments). Approximately 0.5 mL of resin is placed between a 25-mm-diameter steel top plate equipped with a solvent trap and a bottom Peltier plate on the instrument for each test. A loading gap of ca. 1000 μm is used for each test. A storage modulus (*G*′) > 30 kPa indicates the formation of elastomeric gels (degree of precure, *α*_0_ > 0.20), which can be removed from glass molds without any tack on the contact surfaces (Supplementary Fig. 1b). Gels with *G'* < 30 kPa (*α*_0_ < 0.20) are difficult to demold cleanly due to deformation of the viscous resin, resulting in irregular surface defects on the cured specimen post-FP.

### Thermal characterization of resin

The degree of precure (*α*_0_) of the resin is tracked during incubation at 20 °C using differential scanning calorimetry (DSC) in a Discovery DSC Q250 (TA Instruments) equipped with an RCS 90 cooling system. Approximately 2–3 mg of resin is placed in an aluminum hermetic DSC pan and sealed before loading onto the DSC autosampler. Loading a small mass of each sample ensures a constant temperature gradient in the instrument despite the highly exothermic nature of the curing reaction. Each sample is subjected to a constant ramp rate of 7 °C min^−1^ between −50 and 250 °C to determine the thermal cure profile (Supplementary Fig. 1a). The baseline-corrected exothermic peak in the heat flow signal is integrated to extract the enthalpy of reaction (*H*_r1_). The degree of precure of each sample is then calculated using Eq. 1:

1 $${\alpha }_{0}=1-\frac{{H}_{{\rm{r}}1}}{{H}_{{\rm{r}}0}}$$

where *H*_r0_ is the enthalpy of reaction of freshly prepared liquid (uncured) resin and *H*_r1_ is the remaining enthalpy of gelled (partially cured) resin. Degree of precure (*α*_0_) of the DCPD resin for different incubation times is summarized in Supplementary Table 1. The specific heat capacity of the matrix (*C*_p1_) is averaged between 20 and 200 °C after calibration with a sapphire standard.

### Melt processing of sacrificial templates

Melt-blending of PPC with PAG is performed below 160 °C to minimize acidic depolymerization of PPC through thermal activation of the PAG during processing. Neat PPC pellets are blended with 1–3% PAG in a twin-screw melt compounder (Type Six, C.W. Brabender^®^ Instruments). The twin-screw chamber is heated to 140 °C after which 40 g of PPC pellets are added to the chamber while rotating the mixing blades at 15 RPM until completely melted (ca. 10 min). The desired amount of PAG powder (per wt. % of PPC) is slowly added and then mixed at 45 RPM for 5 min. The melt-compounded material is removed from the chamber, cut into small pieces while still soft, then allowed to cool to RT.

Sacrificial fibers and filaments are produced using a modified lab-scale melt-spinning apparatus (extruder). The blended polymer (20 g) is fed into a steel barrel and melted at 155 °C for 10 min. A polytetrafluoroethylene (PTFE) disc followed by a brass-capped steel piston is inserted into the top opening of the barrel to prevent the melted polymer from sticking to the piston. The sacrificial polymer is extruded through a 1.25 mm diameter spinneret at 3 g min^−1^ by mechanically advancing the piston. A metal pulley translates laterally and guides the extruded fiber to wrap uniformly around a spool mounted on an 88-mm diameter winding reel. The surface speed of the take reel is adjusted to control the final diameter of the fiber. For example, a surface speed of 120 m min^−1^ produces roughly 200 m of 400 μm diameter fiber. The sacrificial filaments are extruded through the same apparatus under the same temperature settings by replacing the smaller spinneret with a 2.5-mm diameter spinneret connected to a 75-mm-long hollow brass extension. The extruded filament (ca. 2.85 mm diameter) enters a 1 m tall water column at RT instead of winding on a take-up drum. The filaments are vacuum dried at 40 °C for 24 h before feeding into the 3D printer.

An *Impatiens* leaf is scanned and its digital image is imported into ImageJ for mapping the key coordinates of the primary and secondary veins (Supplementary Fig. 12). The coordinates are then converted into a g-code and loaded into a TAZ6 fused deposition modeling printer (Lulzbot). The spun PPC (1% PAG) printing filament is fed into the printer and extruded at 160 °C through a 500 μm diameter nozzle onto an 80 °C bed. The template is printed at a 0.4 mm print height and 35 mm min^−1^ material feed rate.

### Photoactivation of acid catalyst

Activation of the PAG is carried out on an IntelliRay UV0320 parabolic flood curing system (Unitron International). Sacrificial fibers (15 cm long and 400–600 µm diameter) or printed templates are placed on an aluminum foil and taped at the ends to prevent shrinking during irradiation. The foil is secured on an adjustable metal shelf 75 mm below the lamp such that all the fibers are within the area projected by the lamp. The fibers are irradiated for a total of 10 min at 100% power (6–8 J cm^−2^ at 280 nm). The templates are flipped after 5 min to promote activation of the PAG through the thickness of the sacrificial polymer. The templates are either used immediately after irradiation or stored at −20 °C in a lightproof box until use. The UV irradiation time for the templates is not optimized and a more powerful UV source would be ideal for activating the PAG rapidly within seconds. Unless otherwise specified, all references to the sacrificial fibers or templates refer to UV-irradiated material.

### Characterization of sacrificial templates

Scanning electron micrographs (SEM) of the fibers are acquired on a FEI Quanta FEG 450 Environmental SEM. Samples are imaged at 5 kV after sputter coating with gold/palladium for 70 s (ca. 7 nm) using a Denton Desk II TSC-turbo-pumped sputter coater.

Molecular weights of pellets and fibers are obtained via analytical gel permeation chromatography (GPC) on a system composed of a Waters (1515) Isocratic high-pressure liquid chromatography pump, a Waters (2414) Refractive Index Detector, a Waters (2707) 96-well autosampler, and a series of 4 Waters HR Styragel columns (7.8 × 300 mm, HR1, HR3, HR4, and HR5) in tetrahydrofuran (THF) at 30 °C. The GPC is calibrated using monodisperse polystyrene standards. Each sample (10 mg) is weighed on an analytical balance (XPE205, Mettler-Toledo) and dissolved in 1 mL THF. The polymer solution is injected into a 300 μL GPC vial through a 0.45 μm polytetrafluoroethylene (PTFE) filter to remove any undissolved particulates before loading it into the GPC autosampler. Number- and weight-averaged molecular weights (*M*_n_ and *M*_w_) are calculated using Wyatt’s Astra 6 software through the integration of the signal peaks obtained from the RI detector (Supplementary Fig. 2a).

Thermal depolymerization via mass loss of sacrificial templates (3 mg samples) in a nitrogen environment was measured on a TGA (Q500, TA Instruments) equipped with an evolved gas analysis furnace and calibrated with nickel standards (Supplementary Fig. 2b, c). For dynamic tests, the mass loss is recorded during a heating cycle over the temperature range from 40–250 °C at a linear ramp rate of 1–100 °C min^−1^. Instrument limitations caused a nonlinear increase in initial temperature from 40 to 100 °C for high ramp rates (>40 °C min^−1^). For isothermal tests, the temperature is ramped from 40 to 10 °C below the desired temperature at a linear ramp rate of 10 °C min^−1^, then subsequently ramped to the desired temperature at a slower linear rate of 5 °C min^−1^ to minimize temperature overshoot. The isothermal temperature is maintained for 3–6 h for each test.

The glass transition temperature (*T*_g_), heat capacity (*C*_p2_), and enthalpy of depolymerization (*H*_r2_) of PPC fibers are measured on the DSC Q250 (Supplementary Fig. 6c, d). Approximately 2–3 mg of the sacrificial polymer is sealed in an aluminum hermetic DSC pan and exposed to a constant ramp rate of 5 °C min^−1^ between −50 and 180 °C. The specific heat capacity of the fibers is averaged between 20 and 140 °C after calibration with a sapphire standard. The *H*_r2_ of the sacrificial polymer is extracted by integrating the baseline-corrected endothermic peak in the heat flow signal between 50 and 180 °C. For open-pan DSC experiments, a TA instrument Q20 DSC connected to a CFL-50 cooling system is used. The enthalpy of vaporization of PC monomer (integrated between 60 and 150 °C) is subtracted from the enthalpy of vaporization of PPC (1% PAG) fiber to estimate the enthalpy of depolymerization of the fiber.

### Specimen fabrication for vascularized neat polymer

For FP of specimens in glass molds, a single PPC (1% PAG) fiber is clamped between two polyurethane spacers (inner dimensions: 60.0 mm × 34.0 mm × 6.0 mm) and sandwiched between two 76.2 mm × 50.8 mm × 6.4 mm glass plates in a cell casting mold (Supplementary Fig. 3). Liquid resin is poured into each mold to completely submerge the fiber. A resistive wire perpendicular to the fiber orientation is then placed in the resin. Resin-filled molds are incubated in an environmental test chamber (MicroClimate, Cincinnati Sub-Zero Products) at 20 °C to achieve the desired degree of precure (*α*_0_) (Supplementary Table 1). Once the desired *α*_0_ is reached, FP of the gelled sample is initiated by briefly powering (6 W for ca. 3 s) the resistive wire.

For FP of specimens without the glass mold, the precured elastomeric gel specimens are removed from the cell casting mold once *α*_0_ > 0.20. The edges of the specimens are trimmed with a razor blade to expose the transverse ends of the sacrificial fiber. FP is then initiated by applying a soldering iron to one end of the specimen.

For the helical microchannel in Fig. 3a, b, a PPC fiber (1% PAG) is heat-set at 40 °C into the desired shape before embedding in the glass mold and filling with resin.

For dynamic modulation experiments in Fig. 3d–f, DCPD gels containing a single sacrificial fiber (located at 1 mm from the surface of a 4-mm-thick sample) are fixed between tensile grips on an Instron 8841 fatigue frame with a gauge length of 50 mm. FP is thermally initiated using a soldering iron at one end of the sample, and dynamic (sinusoidal) loading is applied as the front propagates. Front propagation, applied tension, and fiber orientation are all in the same direction. The displacement amplitude is maintained at 4 mm for all samples and the frequency is varied from 0.1 to 1 Hz.

For the leaf specimen in Fig. 4, the PPC (1% PAG) template was submerged in liquid DCPD resin inside a glass mold that was subsequently incubated at RT for ~12 h prior to initiating FP. The gelled specimen (*α*_0_ ~ 0.25) was removed from the mold and a soldering iron was used to trigger the FP on one side perpendicular to the primary vein of the template.

### Microchannel characterization and reconstruction

The vasculature is rinsed with ethanol after FP and optical images of polished cross sections are captured on a Keyence VHX-5000 digital microscope at 100×-200× magnification. Microchannels within the host matrices are also visualized with X-ray computed microtomographic (μCT) imaging (Xradia MicroXCT-400 using TXM Controller software). The μCT scans are conducted after infiltrating the empty microchannels with a contrast solution (350 mg Histodenz^TM^ in 1 g DI water). For 5-mm long channels, 360° scans are obtained in rotation intervals of 0.4° with a ×4 objective (5 μm per pixel) at 5 s exposure times with 40 kV (200 μA, 8 W) source. For 20-mm long microchannels, imaging settings are changed to rotation intervals of 0.5° with a ×1 objective (20 μm per pixel) at 3 s exposure times with 60 kV (133 μA, 8 W) source. Scan reconstructions are completed in a proprietary software and images are produced via Amira^TM^ (v. 6.4.0). ImageJ software is used to measure the dimensions and circularity of the channels from optical images and μCT reconstructions (Supplementary Table 5).

### Computational modeling of coupled polymerization and vascularization

The frontal polymerization of DCPD and the depolymerization of PPC are coupled thermochemical processes described by their own reaction-diffusion model. For computational efficiency, we simulate the PPC fiber embedded in a cylindrical DCPD domain (Supplementary Fig. 7a) in an axisymmetric setting. In the DCPD domain, the thermochemical model expressed in terms of the temperature, *T* (in K), and degree-of-cure, $\alpha$, takes the form

2 $$\left\{\begin{array}{c}{\hskip-1pc\kappa }_{1}\frac{{\partial }^{2}T}{\partial {r}^{2}}+{\kappa }_{1}\frac{1}{r}\frac{\partial T}{\partial r}+{\kappa }_{1}\frac{{\partial }^{2}T}{\partial {z}^{2}}+{\rho }_{1}{H}_{{\mathrm{r}}1}\frac{\partial \alpha }{\partial t}={\rho }_{1}{C}_{{\mathrm{p}}1}\frac{\partial T}{\partial t}\\ \frac{\partial \alpha }{\partial t}={A}_{1}\exp \big(-\frac{{E}_{a1}}{\mathrm{RT}}\big){(1-\alpha )}^{{n}_{1}}{\alpha }^{{m}_{1}}\frac{1}{1+\exp [C(\alpha -{\alpha }_{\mathrm{c}}-{\alpha }_{0})]}\end{array}\right.$$

where the subscript “1” denotes quantities associated with DCPD, *α* = 0 denotes the uncured resin, and *α* = 1 denotes the fully cured polymer. In (2), *κ* (in W m^−2^ K^−1^) denotes the thermal conductivity, *ρ* (in kg m^−3^) the density, *C*_p_ (in J g^−1^ K^−1^) the specific heat, and *H*_r_ (in J g^−1^) the total enthalpy of reaction. The second equation in (2) captures the cure kinetics, with *A* (in s^−1^) denoting the pre-exponential constant, *E*_a_ (in kJ mol^−1^) the activation energy, and *R* (= 8.314 J mol^−1^ K^−1^) the universal gas constant. The additional terms appearing in the cure kinetic model are based on the Prout–Tompkins auto-catalytic model^41^ ${(1+\exp [C(\alpha -{\alpha }_{\mathrm{c}}-{\alpha }_{0})])}^{-1}$, which also accounts for *α*_0_. This model has been successfully used to simulate FP in DCPD monomer^42^ and DCPD-based composites^43–45^. Supplementary Table 3 provides the material properties and Supplementary Table 4 provides the cure kinetic parameters for DCPD.

The depolymerization process of the PPC fiber is described similarly in terms of the thermal field and the degree of depolymerization $\beta$, with $\beta =0$ corresponding to the intact PPC and $\beta =1$ the fully depolymerized state:

3 $$\left\{\begin{array}{c}{\kappa }_{2}\frac{{\partial }^{2}T}{\partial {r}^{2}}+{\kappa }_{2}\frac{1}{r}\frac{\partial T}{\partial r}+{\kappa }_{2}\frac{{\partial }^{2}T}{\partial {z}^{2}}-{\rho }_{2}{H}_{{\mathrm{r}}2}\frac{\partial \beta }{\partial t}={\rho }_{2}{C}_{{\mathrm{p}}2}\frac{\partial T}{\partial t}\\ \hskip-4pc \frac{\partial \beta }{\partial t}={A}_{2}\exp\, \big(-\frac{{E}_{{\mathrm{a}}2}}{\mathrm{RT}}\big){(1-\beta )}^{{n}_{2}}{\beta }^{{m}_{2}}\end{array}\right.$$

where the subscript “2” denotes quantities associated with PPC, and *H*_r2_ the total heat absorbed per unit mass by the depolymerization of the sacrificial material. To extract the parameters defining the depolymerization kinetics of PPC, a constrained nonlinear multi-variable optimization algorithm^46^ is adopted to match dynamic TGA data obtained at different ramping rates (Supplementary Fig. 6a, b). The material properties and depolymerization kinetics parameters used in this study for PPC are listed in Supplementary Tables 3 and 4, respectively.

As shown in Supplementary Fig. 7b, convective boundary conditions (with a film coefficient *h* and an ambient temperature of 20 °C) are applied along the outer surfaces of the cylindrical domain. In the simulation, the DCPD radius (*r*_1_) is 3 mm, the fiber radius (*r*_2_) is 200 μm, and the length of the domain (*l*) is 20 mm. Initiation of the FP process is achieved by prescribing a temperature (200 °C) for one second along the edge at *x =* 0. The film coefficient is chosen (*h =* 25 W m^−2^ K^−1^) such that the temperature profile in the DCPD is similar to that measured in the experiments. For the simulations involving a glass mold, a glass layer is added along the top of the simulation domain with a glass thickness of 0.7 mm to capture the temperature history (Supplementary Fig. 7c). In that glass layer, the transient heat conduction equation (in the absence of the reaction term) is solved, with the convective boundary condition applied to the surface of the glass layer exposed to air.

The analysis is performed with Multiphysics Object-Oriented Simulation Environment (MOOSE), an open-source C++ math library and nonlinear finite-element solver^47^ that provides the mesh adaptivity needed to capture accurately and efficiently the sharp gradients in temperature and degree of cure/depolymerization in the vicinity of the advancing front.

### Specimen fabrication for vascularized fiber-reinforced composites

Composites specimens are fabricated with 10 plies of 2 × 2 twill weave carbon-fiber fabric. The DCPD resin solution is prepared with 0.3 equivalents of TBP with respect to GC2. A wet layup technique is used to infuse the liquid DCPD resin into a 10 cm by 13 cm stack of dry carbon-fiber fabric with a PPC fiber (3% PAG, 400 μm diameter) secured between the fifth and sixth layer (midplane of the fabric stack) (Supplementary Fig. 11). The layup is prepared on a resistive heater (OMEGALUX®), which is secured onto an insulator tool plate (448-D, Fibre Glast Developments Corp.). Silicone spacers (4-mm thick) are placed adjacent to the layup to dictate the cured composite thickness. An additional insulator tool plate is rested on top of the layup, then the entire setup is moved to a hydraulic press (MTP-13, Tetrahedron). A platen force of 2 kN is applied onto the setup and then FP is initiated by powering the resistive heater. The heater was switched off as soon as a spike in the thermocouple temperature was observed (ca. 30 s) indicating that the front has propagated through the composite thickness (Supplementary Fig. 1d).

Depolymerization of the sacrificial fiber post-FP is evaluated by passing a steel wire through the channel. The channel fidelity and void content in the cured composite are measured by polishing the cross sections of samples cut along the panel, then imaging the polished surfaces via a digital optical microscope (VHX-5000, Keyence). ImageJ software is used to measure the circularity of the channel and to calculate the ratio of void to cross-sectional area for each polished sample. The composite fiber volume fraction, *V*_f_, is calculated using Eq. 4:

4 $${V}_{{\rm{f}}}=\frac{{f}_{{\rm{A}}}n}{{\rho }_{{\rm{f}}}z}$$

where *f*_A_ is the areal weight of the fabric, *ρ*_f_ is the fiber density, *n* is the number of plies, and *z* is the composite thickness.

### Energy and time consumption calculations

The energy consumed during any heating step is determined by multiplying the energy consumption rate by the step time. The energy consumption rate during ramp and dwell steps for an oven with a power rating of 4.8 kW and an interior volume of 0.8 m^3^ were 0.046 and 0.024 kWh min^−1^, respectively^36^. The following protocols were used for each process:

BPA-epoxy oven-cure cycle: ramp from 20 to 121 °C (50 min), dwell at 121 °C (2 h), ramp to 177 °C (25 min), and dwell at 177 °C (3 h).

DCPD oven-cure cycle: ramp from 20 to 70 °C (30 min), dwell at 70 °C (2 h), ramp to 170 °C (50 min), and dwell at 170 °C (1.5 h).

PLA VaSC cycle: ramp from 20 °C to 200 °C (2 h) and dwell at 200 °C (12 h).

DCPD FP initiation with resistive wire: A DC source with 3 A at 2 V was switched on for 3 s.

FRPC FP initiation with heating mat: A 300 W heater was powered on for 30 s.

UV irradiation cycle for PPC: 10 min at 10 mW cm^−2^ for a 15 cm long fiber.

## Supplementary information

Supplemental Information

Description of Additional Supplementary Files

Supplementary Video 1

Supplementary Video 2 

Supplementary Video 3

Supplementary Data 1

## Data Availability

Source data are provided with this paper. Any supplementary data that support the findings of this study are available from the corresponding author on reasonable request.

## Code availability

We performed the simulations by developing an application based on MOOSE, an open-source C++ finite-element framework developed at Idaho National Laboratory. MOOSE is freely available at mooseframework.org. A compiled version of the code for simulating the synchronized manufacturing process, an example input file, and the instructions for running the simulations are provided and can be accessed under the MIT License at https://gitlab.com/zhangx22/concurrentvascfp. These files could be used to reproduce the computational results reported in this manuscript or simulate the tandem polymerization and depolymerization processes with a different geometrical settings, initial or boundary conditions.
