# Supplementary material for: Rapid synchronized fabrication of vascularized thermosets and composites
Source: Nat Commun. 2021 May 14;12:2836. doi: 10.1038/s41467-021-23054-7 (PMC8121863; doi:10.1038/s41467-021-23054-7)
Supplement: Supplementary file 1 — Supplemental Information [file 41467_2021_23054_MOESM1_ESM.pdf]

Supplementary Information

Rapid Synchronized Fabrication of Vascularized Thermosets and Composites

Mayank Garg, Jia En Aw, Xiang Zhang, Polette J. Centellas, Leon M. Dean, Evan M. Lloyd, Ian D. Robertson, Yiqiao Liu, Mostafa Yourdkhani, Jeffrey S. Moore, Philippe H. Geubelle, *Nancy R. Sottos

Thermal and Rheological Characterization of Matrix

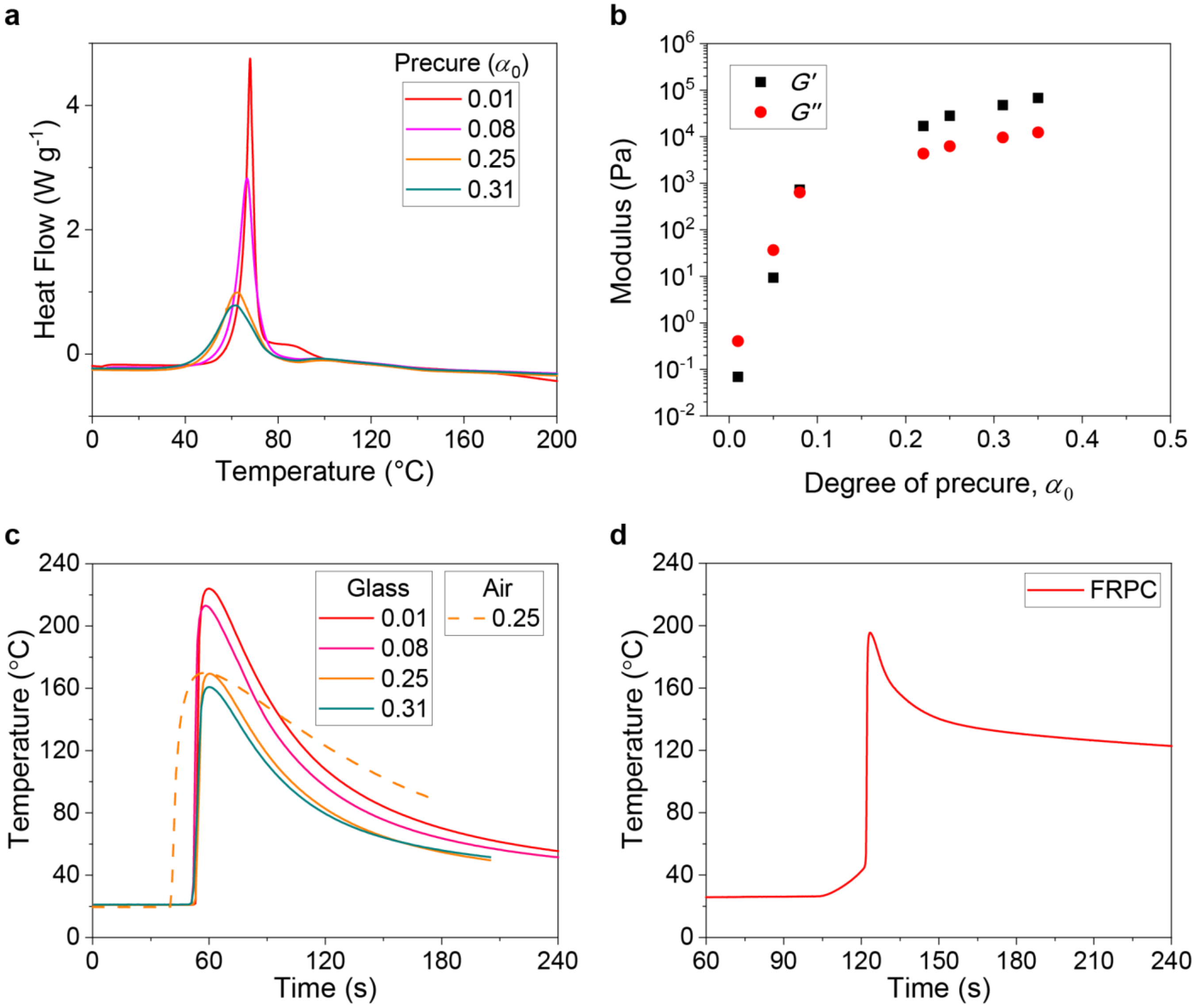

Supplementary Fig. 1 Characterization of DCPD resin and fiber-reinforced polymer composite (FRPC). a DSC scans of DCPD incubated for different time intervals at 20 °C to determine degree of precure (α_0). Correlation between the precure and incubation time is listed in Supplementary Table 1. **b** Storage (*G*') and loss (*G*") moduli of DCPD resin incubated at 20 °C under oscillatory shear of 1 Hz. The G' and G" crossover occurs at α_0 ~ 0.08. **c** Temperature profile during FP of DCPD specimens with varying α_0 for glass boundaries (solid lines) and without glass boundaries (dashed line). **d** Temperature profile of fiber-reinforced polymer composite (FRPC) during FP.

Characterization of Melt-Spun Sacrificial Fibers

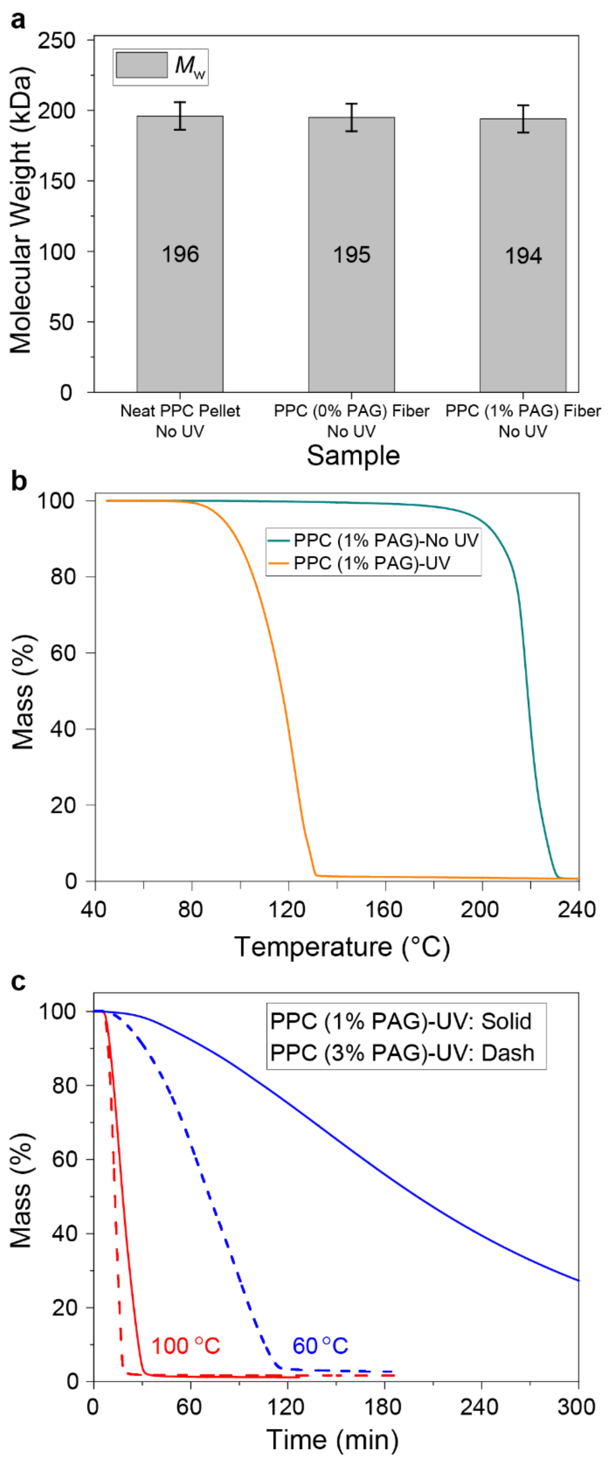

Supplementary Fig. 2 Molecular weight and mass loss characterization of melt-spun sacrificial fibers. a Gel permeation chromatography (GPC) of PPC pellets and fibers shows no significant difference in weight-averaged molecular weight due to thermal latency of the PAG during fiber spinning. Error bars represent one standard deviation (*n* =3). **b** Thermal depolymerization of PPC fibers in dynamic thermogravimetric analysis (TGA). UV-irradiated fibers depolymerize at significantly lower temperatures than as-spun fibers. **c** Mass loss of UV-irradiated fibers under isothermal TGA conditions.

Demonstration of Successful and Unsuccessful Vascularization

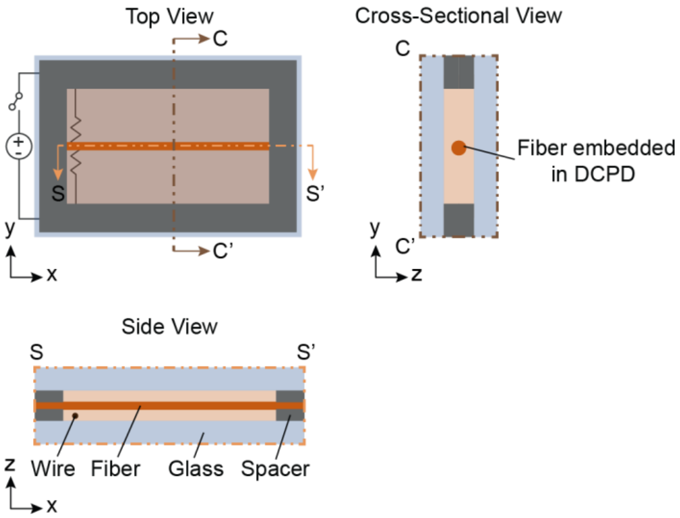

Supplementary Fig. 3 Cell casting of sacrificial fibers in DCPD resin. The sacrificial PPC fiber (orange) is clamped by two rubber spacers (dark grey), then sandwiched between two glass plates (light blue). Liquid resin (light orange) is poured in the cell casting mold to submerge the fiber.

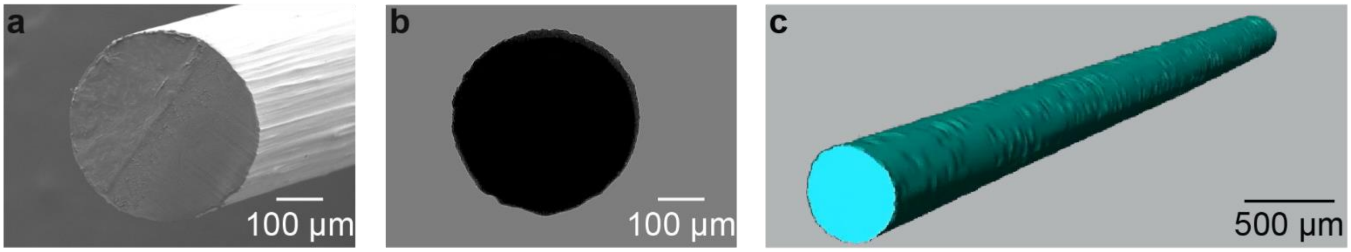

Supplementary Fig. 4 Images of sacrificial fiber and microchannel in pDCPD matrix. a SEM micrograph of an as-spun PPC (1% PAG) fiber. **b** Optical micrograph of a microchannel in pDCPD matrix after concurrent FP and VaSC of a DCPD gel (α_0 = 0.25) containing a PPC (1% PAG, UV-irradiated) fiber. **c** X-ray computed microtomographic (µCT) reconstruction of the vascular pDCPD sample reveals a cylindrical microchannel with high fidelity to the initial PPC fiber.

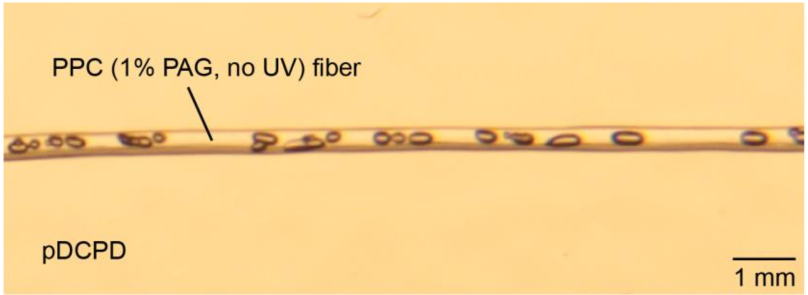

Supplementary Fig. 5 Unsuccessful vascularization of sacrificial fiber. The PPC (1% PAG) fiber without UV irradiation does not depolymerize during FP of a DCPD gel (α_0 = 0.25), preventing the formation of microchannels.

Coupled Thermochemical Model

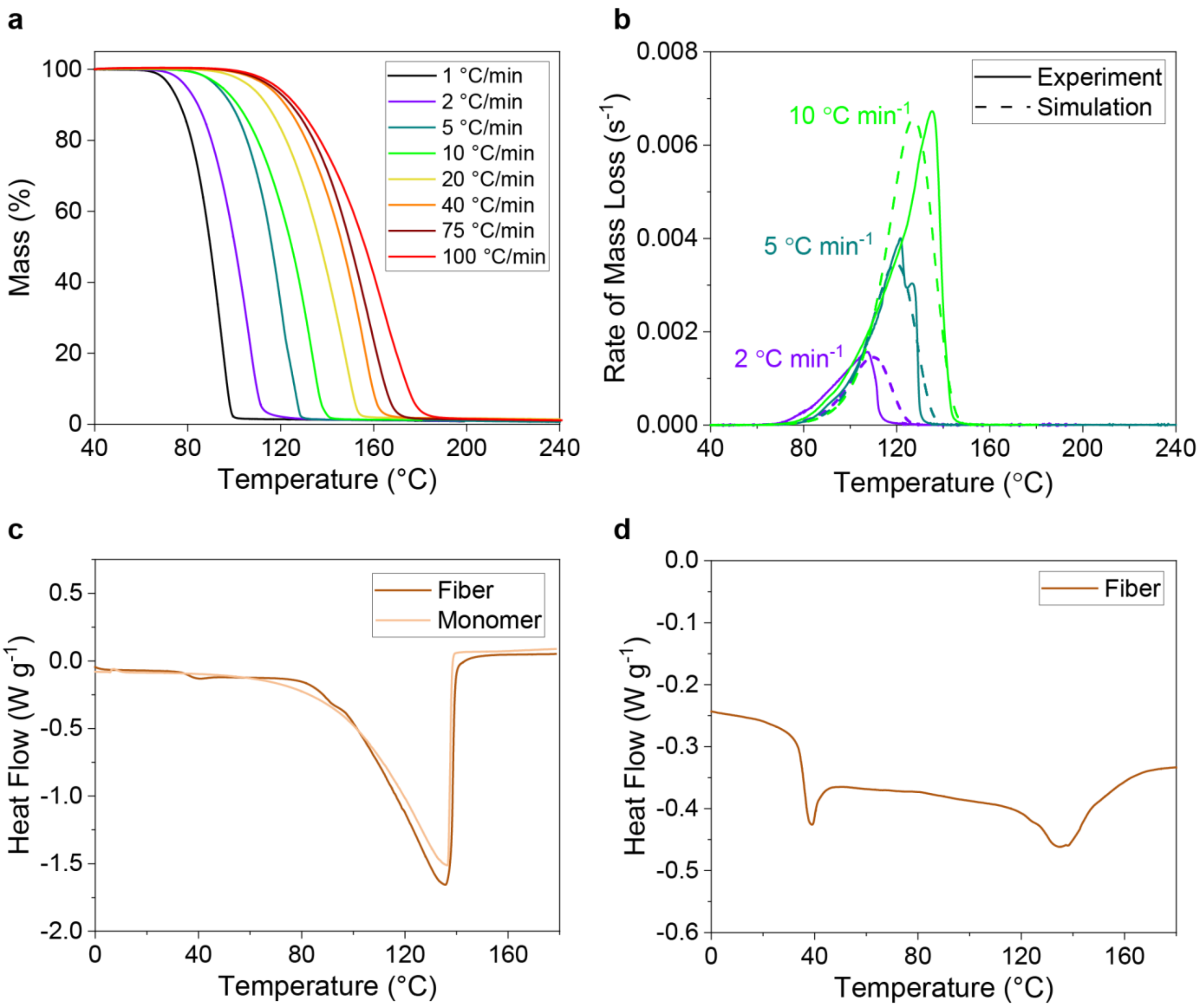

Supplementary Fig. 6 Modeling depolymerization of UV-irradiated PPC (1% PAG) fibers. a Dynamic TGA of fibers at several ramp rates. **b** Depolymerization kinetics model using dynamic TGA data at three different ramp rates. **c** DSC of PPC and PC monomer at 5 °C min^{-1} in open-pan setting. The heat of vaporization of PC (556 J g^{-1}) matches reported values[1] and an additional H_{r2} = 19 J g^{-1} is consumed during PPC depolymerization. **d** DSC of PPC at 5 °C min^{-1} in closed-pan configuration which suppresses vaporization. The T_g of PPC is ca. 38 °C and total heat absorbed by the fiber between 50 and 180 °C amounts to 21 J g^{-1}, which is close to the calculated H_{r2} from open-pan tests.

Experiments vs. Simulations

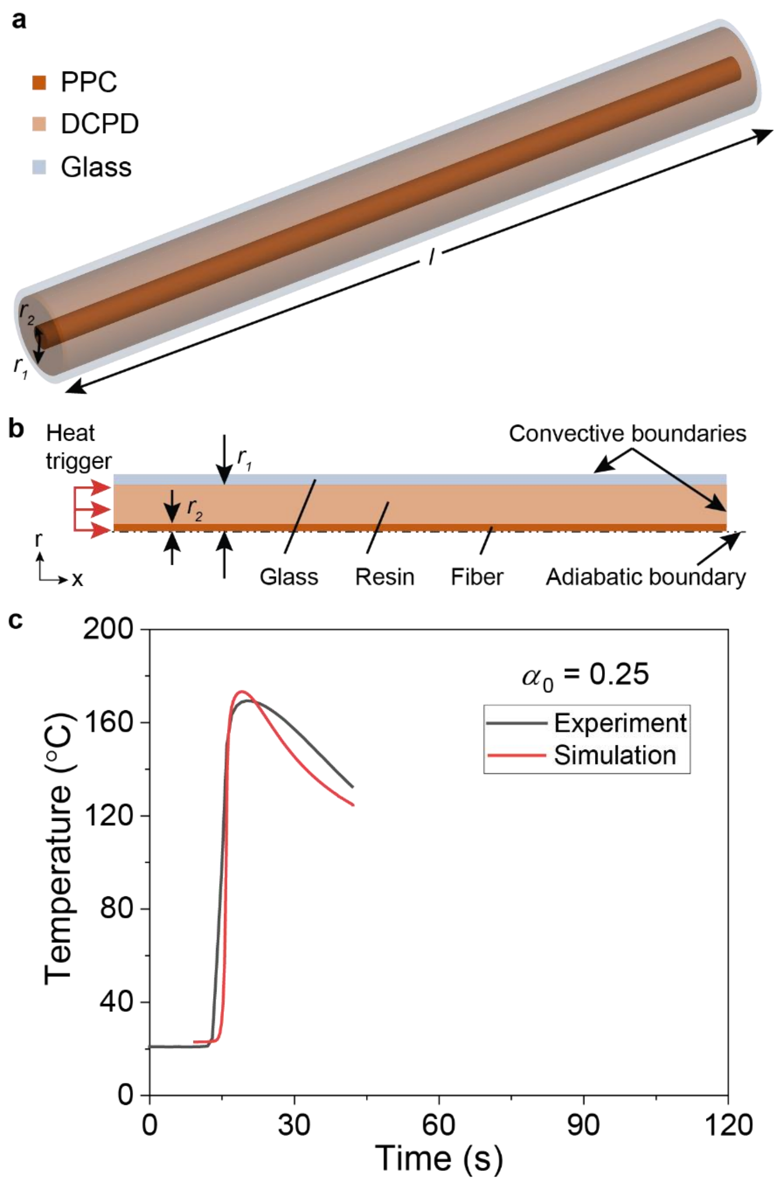

Supplementary Fig. 7 Simulation domain for coupled polymerization and vascularization. a A single PPC fiber, with radius r_2 and length l, is embedded in cylindrical DCPD matrix, with radius r_1 and length l. **b** Corresponding 2D axisymmetric domain used in the simulation. For the glass boundary case, a layer of glass is added onto the DCPD, and the convection boundary is then applied on the outer surface of the glass. **c** Thermal profile comparison between experiments and simulations. A 0.7 mm thick glass plate and a convective film coefficient of 25 W m^{-2} K^{-1} in the 2D simulation domain are used to predict a cooling profile for α_0 = 0.25 that closely matches experiments. The temperature predictions are extracted at a vertical distance of 1 mm from the center of the domain.

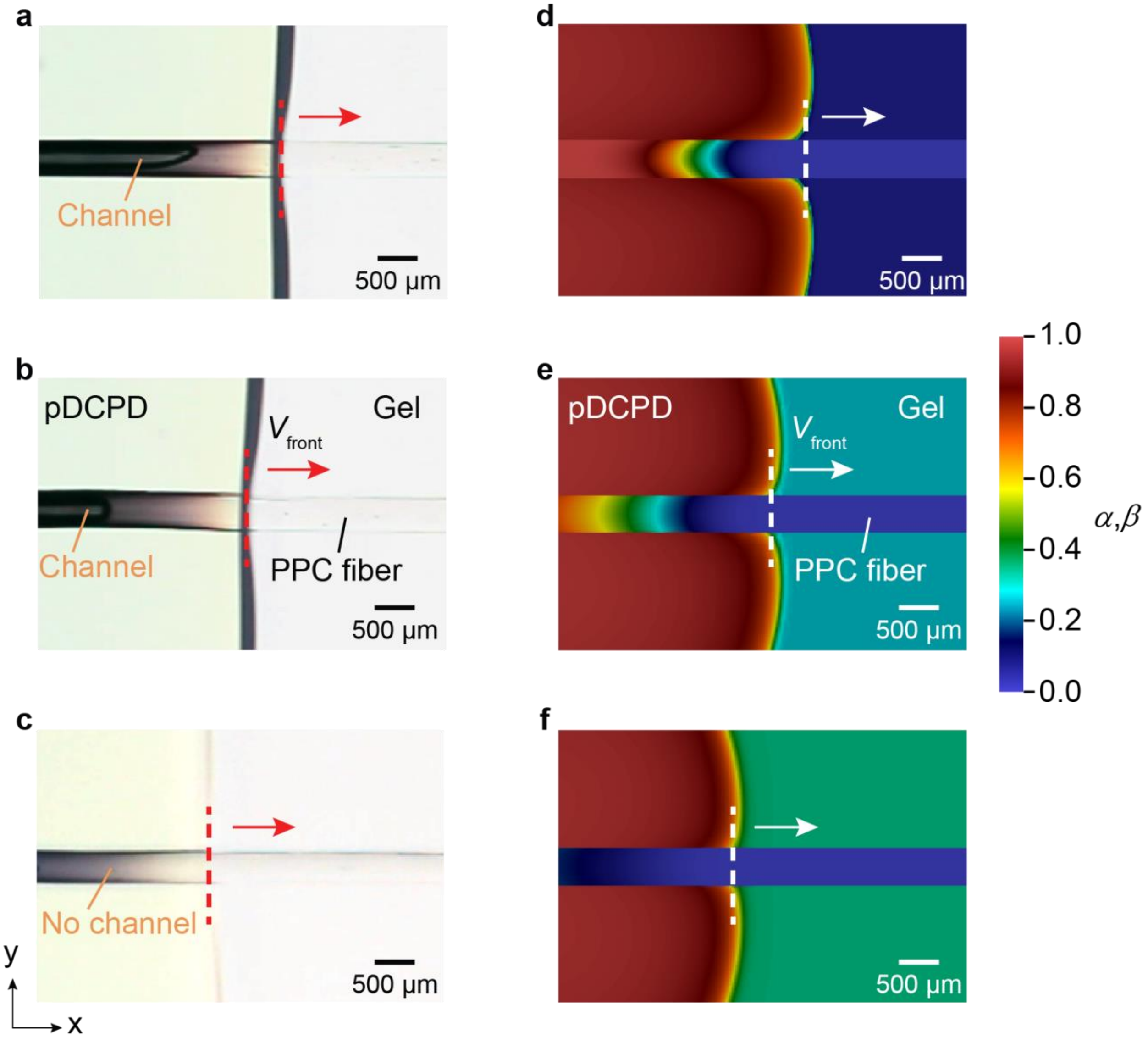

Supplementary Fig. 8 Experimental (left) and simulation (right) snapshots during concurrent polymerization and vascularization of DCPD gels inside glass molds. The experimental images (**a, b, c**) are acquired from videos of the coordinated frontal polymerization/depolymerization process after the front has reached a steady state for specimens with varying α_0. Successful vascularization via complete depolymerization of PPC (1% PAG) fibers was observed for samples with α_0 = 0.08 (**a**) and α_0 = 0.25 (**b**). Obstructed microchannels were obtained for α_0 = 0.35 due to partial depolymerization of the sacrificial fibers (**c**). **d** Simulation prediction for α_0 = 0.08 at t=4 s. **e** Simulation prediction for α_0 = 0.25 at t=31 s. **f** Simulation prediction for α_0 = 0.35 at t=50 s. All times are reported after initiation of FP at t=0 s.

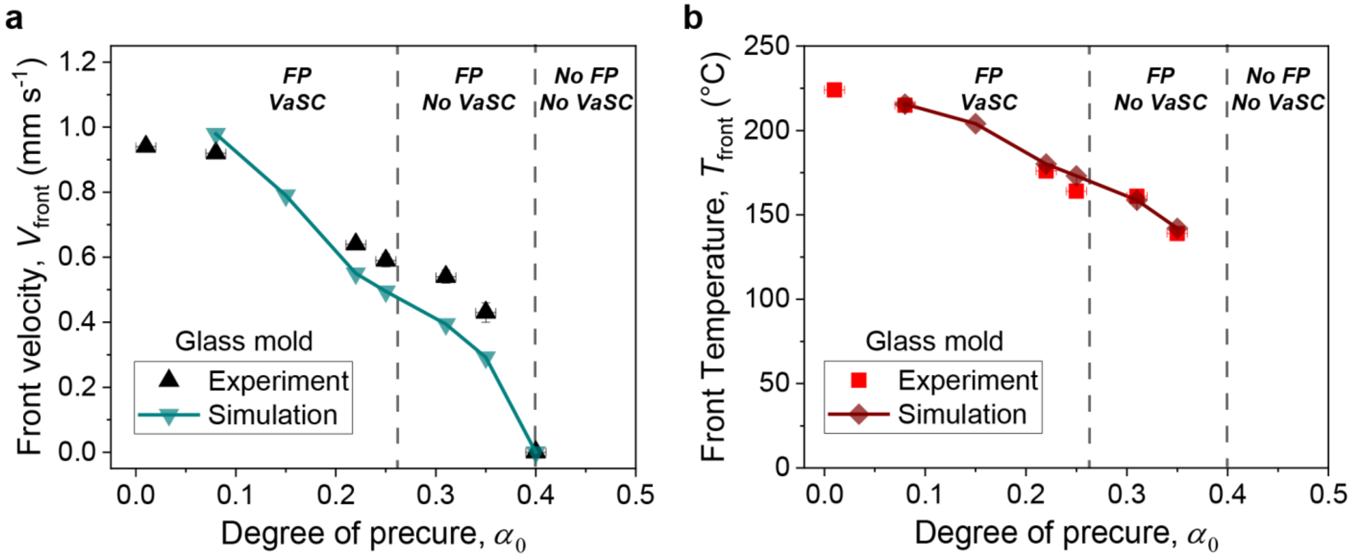

Supplementary Fig. 9 Concurrent polymerization and vascularization window for neat matrix specimens inside glass molds. Experimental measurements compare well to modelling predictions for **a** Front velocity (V_{front}) and **b** Maximum front temperature (T_{front}).

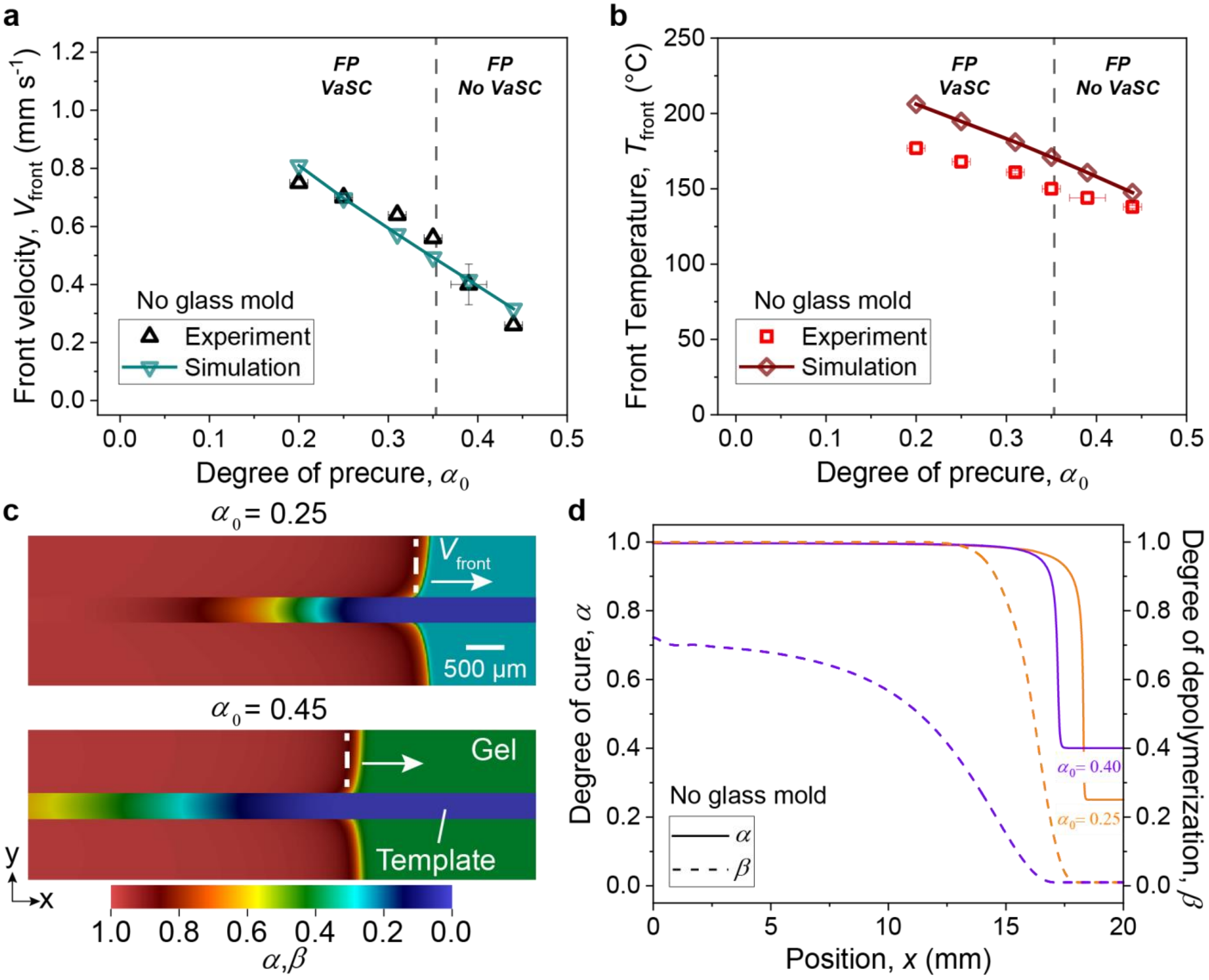

Supplementary Fig. 10 Concurrent polymerization and vascularization window for neat matrix specimens without glass molds. Comparison between **a** experimental front velocity (V_{front}) and **b** maximum front temperature (T_{front}) during FP of DCPD gels with different degrees of precure under convective boundary conditions (RT = 20 °C) show two regimes for successful and unsuccessful vascularization. The enthalpy released during FP is sufficient for depolymerizing PPC (1% PAG) fibers to get unobstructed microchannels up to $\alpha_0 = 0.35$. FP of gels with $\alpha_0 = 0.40$ and 0.45 results in clogged microchannels with partially depolymerized fibers. Error bars represent one standard deviation from the mean (n = 3). **c** Simulation snapshots of DCPD gels showing spatial distribution of the degree of cure (α) of the DCPD matrix and the degree of depolymerization (β) of the sacrificial fiber during FP for α_0 = 0.25 (t=26 s) and $\alpha_0 = 0.40$ (t=41 s). **d** Computational predictions for α and β. The model predicts complete depolymerization for $\alpha_0 = 0.25$ case (orange lines at t=26 s) and partial depolymerization for $\alpha_0 = 0.40$ specimens (purple lines at t=41 s), which is verified experimentally. The time is reported after initiation of FP at t=0 s.

Vascularized Composites

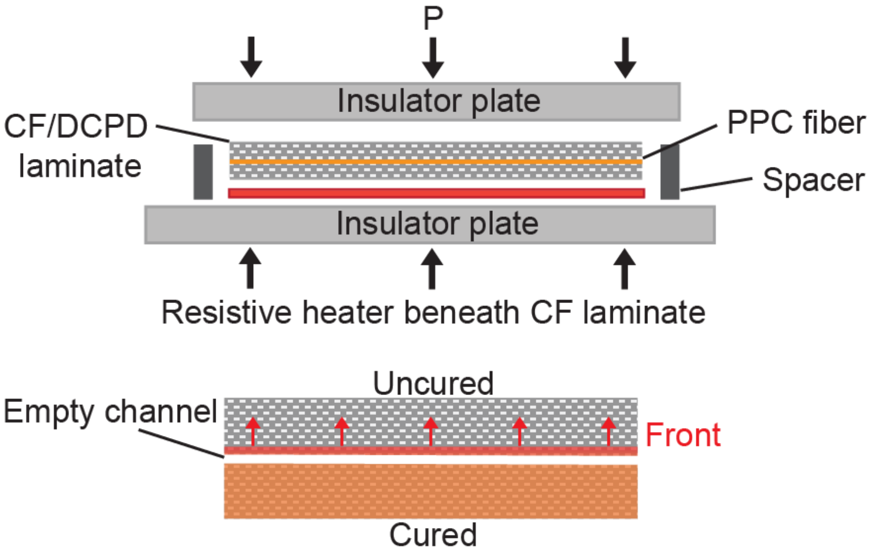

Supplementary Fig. 11 Layup scheme for vascularized fiber-reinforced composites. Side-view schematic of wet layup prior to FP initiation (top row) and schematic of FP curing of carbon fiber (CF) laminate with simultaneous depolymerization of PPC fiber (3% PAG, UV-irradiated) (bottom row) for a through-thickness trigger.

Sacrificial Template Processing for Branched Vasculature

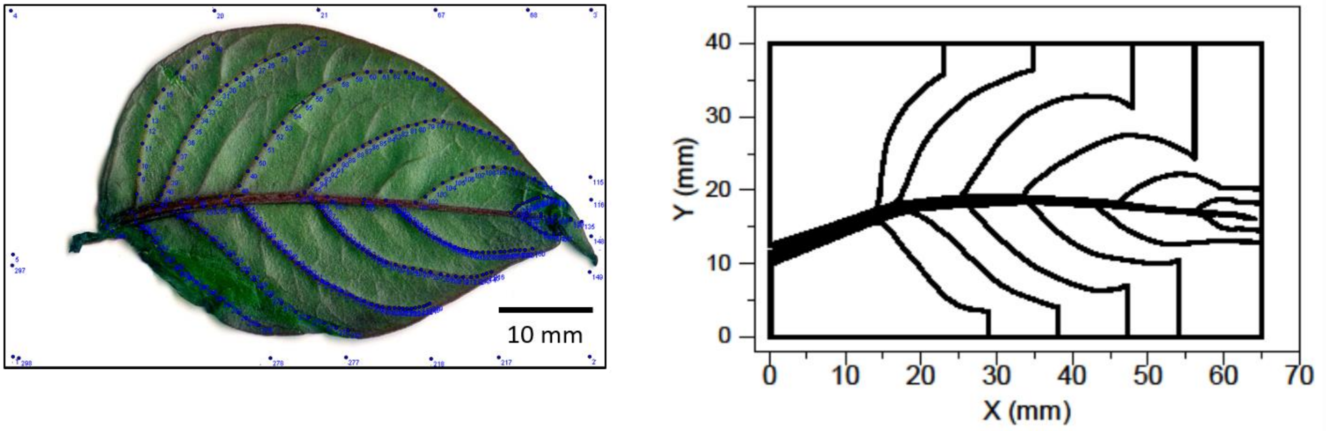

Supplementary Fig. 12 3D printing sacrificial PPC templates (1% PAG). Scanned image of a leaf is used to map the print code coordinates for the leaf veins (left image)**.** Print path of the leaf-like sacrificial template (right image).

Supplementary Table 1 | Degree of precure (α_0) of DCPD resin during incubation at 20 °C.

Incubation Time (min)	Degree of precure (α_0)*
0-30	0.01
450	0.08
720	0.22
780	0.25
840	0.31
900	0.35
960	0.40

*Average degree of precure from 5 samples for each incubation time (standard deviation = 0.01).

Supplementary Table 2 | Energy estimation for conventional curing and then VaSC (two-step) vs. tandem polymerization and vascularization (one-step).

Process	Step	Time (min)	Step Energy (J)	Total Energy (J)
Two-step (A)	BPA Epoxy Oven Cure	375	3.8×10^7	1.20×10^8
	PLA VaSC	840	8.2×10^7	
Two-step (B)	DCPD Oven Cure	290	3.1×10^7	1.13×10^8
	PLA VaSC	840	8.2×10^7	
One-step (DCPD)	FP + PPC (1% PAG) VaSC	1.3	1.08×10^2	1.08×10^2
One-step (FRPC)	FP + PPC (3% PAG) VaSC	0.5	9.09×10^3	9.09×10^3

$$\textit{Energy savings ratio for thermosets} = \frac{\text{Two-step (A)}}{\text{One-step (DCPD)}} = 1.09 \text{ x } 10^6$$

$$\textit{Energy savings ratio for composites} = \frac{\text{Two-step (A)}}{\text{One-step (FRPC)}} = 1.32 \text{ x } 10^4$$

$$\textit{Fabrication time savings ratio for thermosets} = \frac{\text{Two-step (A)}}{\text{One-step (DCPD)}} = \frac{1215}{1.3} = 0.93 \text{ x } 10^3$$

$$\textit{Fabrication time savings ratio for composites} = \frac{\text{Two-step (A)}}{\text{One-step (FRPC)}} = \frac{1215}{0.5} = 2.43 \text{ x } 10^3$$

Supplementary Table 3 | Physical and thermal properties of the various components used in thermochemical computational model.

Material	κ (W m^{-1}K^{-1})	ρ (kg m^{-3})	C_p (J kg^{-1} K^{-1})	H_r (J g^{-1})
DCPD resin	0.15	980	1,600	380
PPC (1% PAG)	0.25	1,313	1,800	20
Glass plate	1.14	2000	800	N/A

Supplementary Table 4 | Kinetic parameters for simulating the degree of conversion of the host and the sacrificial components in the computational model.

Material	E (kJ mol^{-1})	A (s^{-1})	m	n	C	α_c
DCPD resin	113.2	8.5 x 10^{15}	0.78	1.74	15.2	0.36
PPC (1% PAG)	110.0	4.0 x 10^{12}	0.21	0.94	N/A	N/A

Supplementary Table 5 | Dimensions and circularity of microchannels formed by synchronized manufacturing.

Sample	*Template diameter* (μm)	*Channel diameter* (μm)	*Difference in diameter*‡ *(%)*	*Channel circularity**
Neat matrix†	409 ± 17	414 ± 15	1.2	0.89 ± 0.01
Fiber-reinforced composite‡	387 ± 18	375 ± 20	3.1	0.88 ± 0.01

†Error represents one standard deviation for measurements across ten different samples.

‡Error represents one standard deviation for three measurements across one sample.

‡$\% \textit{ difference in diameter} = \frac{|\text{Template diameter} - \text{Channel diameter}|}{\text{Template diameter}} \times 100$

*Circularity of fibers was ca. 0.99 ± 0.01

Supplementary References

1. Hong, C. S., Wakslak, R., Finston, H. & Fried, V. Some thermodynamic properties of systems containing propylene carbonate and ethylene carbonate. *J. Chem. Eng. Data.* **27**, 146–148 (1982).
