# Supplementary material for: Rapid synchronized fabrication of vascularized thermosets and composites
Source: Nat Commun. 2021 May 14;12:2836. doi: 10.1038/s41467-021-23054-7 (PMC8121863; doi:10.1038/s41467-021-23054-7)
Supplement: Supplementary file 2 — Description of Additional Supplementary Files [file 41467_2021_23054_MOESM2_ESM.pdf]

Description of Additional Supplementary Files

File Name: Supplementary Movie 1
Description: Tandem polymerization and vascularization of a DCPD gel ($\alpha 0 = 0.25$) with an embedded sacrificial fiber. FP is triggered in 3 seconds with a resistive heating wire oriented perpendicular to the PPC (1% PAG, UV irradiated) fiber inside a glass mold. The sacrificial fiber depolymerizes during curing of the matrix and a 5 cm long vascular thermoset is manufactured within 2 minutes. The video is presented at 1x speed. The scale bar is 1 mm.

File Name: Supplementary Movie 2
Description: Coordinated polymerization and vascularization of an elastic DCPD gel ($\alpha 0 = 0.25$) with a straight PPC (1% PAG, UV irradiated) fiber under oscillatory loading. The gel is firmly clamped between two grips of an Instron machine and the front is initiated using a linear heat source on the right-hand side (static grip). The specimen is oscillated with a maximum amplitude of 4 mm and frequency of 0.25 Hz from the left-hand side (dynamic grip) during propagation of FP. The temporary deformation of the fiber with the gel is permanently fixed in the sinusoidally-shaped microchannel after FP. The video is presented at 4x speed. The scale bar is 5 mm

File Name: Supplementary Movie 3
Description: Synchronized fabrication of a hierarchical vascular structure with interconnected microchannels. A 3D printed PPC (1% PAG, UV irradiated) template is incubated inside DCPD resin ($\alpha 0 = 0.25$). FP is triggered from the left-hand side with a linear heat source and the template depolymerizes concurrently during propagation of FP. A neat thermoset with interconnected microchannels resembling the primary and secondary veins of an Impatiens leaf is fabricated in 80 seconds. The video is presented at 3x speed. The scale bar is 5 mm.
